# Pilot-Scale Enzymatic Conversion of Low Stability, High Free Fatty, Squid Oil to an Oxidatively Stable Astaxanthin-Rich Acylglyceride Oil Suitable for Nutritional Applications

**DOI:** 10.3390/md23010021

**Published:** 2025-01-02

**Authors:** Asavari Joshi, Brendan Holland, Moninder Sachar, Colin J. Barrow

**Affiliations:** 1 ARC Industrial Transformation Training Centre for Green Chemistry in Manufacturing, Deakin University, Waurn Ponds, Geelong, VIC 3216, Australia; 2 Centre for Sustainable Bioproducts, Deakin University, Waurn Ponds, Geelong, VIC 3216, Australia; brendan.holland@deakin.edu.au; 3 Australian Omega Oils Pty Ltd., North Geelong, Geelong, VIC, 3215, Australia; monindersachar@gmail.com; 4 Distinguished Visiting Research Fellow, College of Engineering, Abu Dhabi University, Zayed City 59911, Abu Dhabi, United Arab Emirates

**Keywords:** enzyme-processed squid oil, rancimat, lipid oxidation kinetics, astaxanthin

## Abstract

Squid viscera, a byproduct of squid processing, contains oil rich in omega-3 fatty acids (up to 10% by mass) and the antioxidant astaxanthin. However, its high free fatty acid (FFA) content compromises stability. To address this, pilot-scale (200 L) enzymatic re-esterification of squid oil using immobilized lipase (Lipozyme RMIM) was demonstrated, resulting in high acylglyceride yields. The processed oil was analyzed for oxidation kinetics and thermodynamics using Rancimat, fatty acid composition using GC, omega-3 fatty acid positional distribution in the acylglyceride product using ^13^C NMR, and astaxanthin content. Lipase treatment reduced FFA levels from 44% to 4% and increased acylglycerides to 93% in squid oil. This reduction in FFA was accompanied by significantly increased stability (0.06 to 18.9 h by Rancimat). The treated oil showed no loss in astaxanthin (194.1 µg/g) or omega-3 fatty acids, including docosahexaenoic acid (DHA). DHA remaining predominantly at sn-2 indicated that the naturally occurring positional distribution of this omega-3 FFA was retained in the product. Lipase treatment significantly enhanced oxidative stability, evidenced by improved thermodynamic parameters (E_a_ 94.15 kJ/mol, ΔH 91.09 kJ/mol, ΔS −12.6 J/mol K) and extended shelf life (IP_25_ 74.42 days) compared to starting squid oil and commercial fish/squid oils lacking astaxanthin. Thus, lipase treatment offers an effective strategy for reducing FFA levels and producing oxidatively stable, astaxanthin-rich acylglyceride squid oil with DHA retained at the nutritionally favored sn-2 position.

## 1. Introduction

Squid cephalopods are an important marine food, while the non-edible parts, such as viscera, are usually discarded [1]. Squid viscera contains high levels of anti-inflammatory marine oils, particularly omega-3 fatty acids, such as docosahexaenoic acid (DHA) and eicosapentaenoic acid (EPA), which account for about 25% of the total lipid content [2]. EPA and DHA have potent anti-inflammatory properties and are precursors to anti-inflammatory mediators such as resolvins and protectins and have been shown to have cardiovascular benefits, be important for normal brain development, and benefit a range of inflammatory mediated conditions [3,4,5]. However, due to their polyunsaturated nature, omega-3 fatty acids are susceptible to auto-oxidation and photosensitized oxidation [6]. The auto-oxidation of these fatty acids leads to free radicals that continue to react during exponential oxidation [7]. The primary products generated during oxidation are called hydroperoxides, which are highly unstable and give rise to the formation of secondary oxidation products, such as ketones, aldehydes, hydrocarbons, alcohols, and acids [7,8,9]. In addition to decreasing omega-3 levels in oil, oxidation causes the formation of sensory, unpleasant aldehyde, making these oils taste bad even after relatively small levels of oxidation [10,11,12]. Squid viscera naturally contains a high level of free fatty acids, most likely due to lipase activity in the gut [13], with high FFA oils being particularly susceptible to oxidation. Squid viscera oil also contains highly colored astaxanthin, making normal colorimetric methods for determining oxidation levels problematic [14].

Astaxanthin is a xanthophyll carotenoid that is found naturally in a variety of marine species, such as shrimp, krill, salmon, squid, and crab, and is a powerful antioxidant [15,16,17,18] that is used in aquaculture, cosmetics, food, feed, nutraceuticals, and pharmaceuticals. Reports have suggested that astaxanthin has the ability to cross the blood-brain barrier, thereby working to reduce free radical-induced neurotoxicity and memory loss, and its anti-inflammatory properties indicate it has cardioprotective benefits and has wound-healing, neuroprotective, hepatoprotective, and osteoprotective properties [18,19,20,21]. The natural form of astaxanthin is normally esterified and has been observed to exhibit higher bioactivity than synthetic astaxanthin [22,23,24,25,26]. Astaxanthin incorporated into omega-3-rich products can provide synergistic antioxidative and anti-inflammatory benefits [27,28,29] and also improve omega-3 oil storage stability [30,31,32,33].

**Figure 1:**
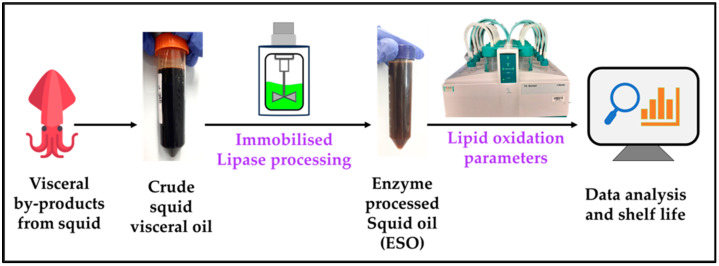
Lipid oxidation parameters of enzyme-processed squid oil using Rancimat.

The Rancimat technique offers accelerated oxidation analysis by measuring volatile oxidation products through electrical conductivity, providing an induction period before accelerated oxidation occurs. It can be used for the rapid determination of oxidative stability and is particularly useful for oils where standard colorimetric methods for oxidation products (peroxide value, PV and p-anisidine values, pAV) are not accurate, such as colored oils or oils containing astaxanthin [34]. The Rancimat technique, including the determination of kinetic and thermodynamic parameters to predict lipid oxidation, has been applied to a range of oils, including vegetable oils [35,36,37,38] and fish oils [39,40,41,42], but only a few studies have investigated non-fish seafood-derived omega-3 oils, such as krill [43], shrimp [15], and squid oil. The current study aims to re-esterify squid visceral oil at the pilot scale (200 L) in order to reduce the levels of free fatty acid content while retaining astaxanthin by employing the in-house enzymatic method [44]. The processed squid visceral oil was then subjected to accelerated oxidation using the Rancimat method to investigate its oxidative stability toward developing this waste oil into a nutritional oil that can be used for nutritional supplements or functional foods. Since there has been no study that has reported kinetic and thermodynamic parameter determination for enzymatically processed squid viscera-derived omega-3-rich oil, lipid oxidation parameters are determined that further aid in understanding the oxidative stability of squid visceral oil. An overview of the study is depicted in Figure 1. The stability of the ESO was then compared with commercially available calamari and fish oil.

The results demonstrate that the reduced free fatty acid content of the oil, together with the retention of natural astaxanthin, produces a more oxidatively stable squid viscera-derived oil compared to other commercially available omega-3 oil sources.

## 2. Results and Discussion

### 2.1. Free Fatty Acid Content in Crude Squid Visceral Oil During Enzymatic Processing

**Figure 2:**
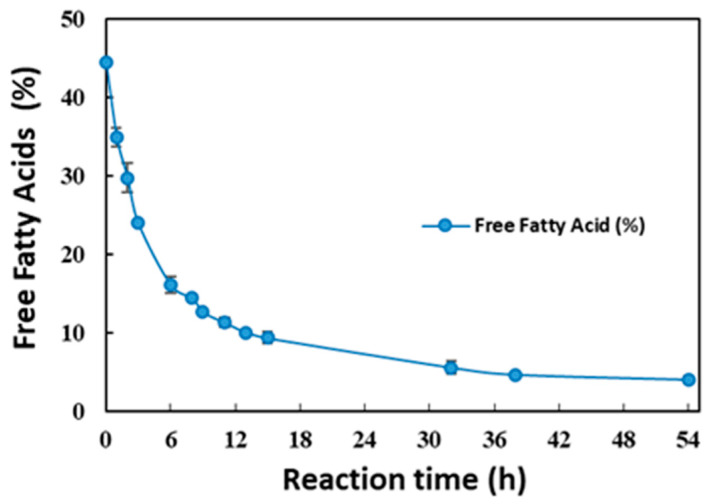
Free fatty acid reduction profile of lipase-processed squid visceral oil (ESO). Results are presented as mean ± SD.

The enzymatic processing of crude squid visceral oil was carried out at the pilot scale using immobilized lipase. The crude squid visceral oil had an initial free fatty acid content of 44 ± 0.39%, which was significantly reduced during the lipase treatment period, as depicted in Figure 2. The free fatty acid content decreased steeply in the initial 9 h, followed by a slow decline to a free fatty acid content of 4 ± 0.5% between 24 h and 54 h. This was attributed to the initial abundance of free fatty acid substrate available for the lipase, resulting in a higher esterification rate during the initial 9 h. As the reaction progressed, the concentration of free fatty acid substrate decreased, leading to a reduced esterification rate beyond 9 h. These findings suggest that future batches of lipase treatment could be halted after 24 h of processing to decrease the reaction time and improve the cost of production.

We determined the lipid class profile of squid visceral oil, including the content of acylglycerides and fatty acid ethyl esters, both before and after lipase treatment. Nutritional oils with a high acylglyceride content exhibit superior stability, sensory acceptance, and bioavailability [45]. In contrast, elevated levels of fatty acid ethyl esters can hinder the digestion and absorption of fatty acids [45] and are associated with an increased risk of arrhythmia and atrial fibrillation [46]. The total acylglyceride content of squid visceral oil increased significantly after lipase treatment from 53.20 ± 0.5% to 93.16 ± 0.5%, while the ethyl ester content remained consistently low, with values of 2.71 ± 0.3% prior to lipase treatment and 2.63 ± 0.2% after lipase treatment. The final product squid visceral oil obtained after pilot-scale lipase treatment consisted of 20.8 ± 1.2% triacylglycerides, 45.6 ± 2.0% diacylglycerides, and 26.8 ± 2.9% monoacylglycerides. This lipid class profile showed a similar trend to the results obtained during the laboratory-scale demonstration in a previous study [44] and to the results reported for the de-acidification of vegetable oil [47]. These findings highlight the potential of immobilized lipase treatment for the large-scale processing of crude squid visceral oil.

### 2.2. Fatty Acid Composition in Crude Squid Visceral Oil During Lipase Processing

The fatty acid composition of the squid oil during enzymatic processing was determined using gas chromatography with a (GC)-flame ionization detector (FID). The results are shown in Table 1.

The results indicate that the fatty acid profile remains relatively stable throughout the enzymatic processing, despite showing statistically significant differences at *p*-value < 0.05. The average total omega-3 (43.0%), eicosapentaenoic acid (20.2%), and docosahexaenoic acid (20.8%) content were higher than those achieved in a previous study [13].

### 2.3. Positional Distribution of Omega-3 Fatty Acids in Lipase-Treated Squid Visceral Oil

**Figure 3:**
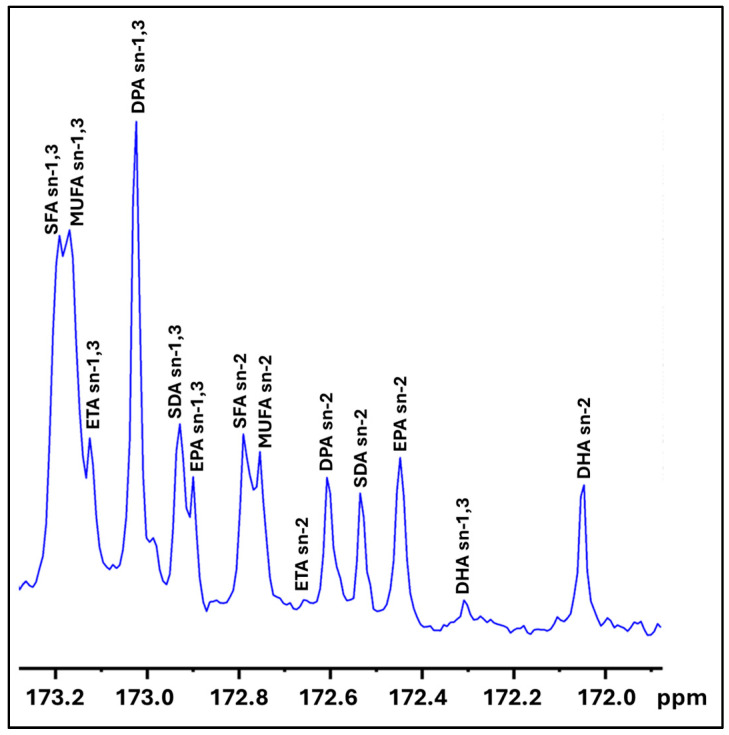
Carbonyl region of ^13^C NMR of lipase-treated squid visceral oil illustrating positional distribution of fatty acids. Abbreviations: DHA (docosahexaenoic acid), EPA (eicosapentaenoic acid), SDA (stearidonic acid), DPA (docosapentaenoic acid), ETA (eicosatetraenoic acid), MUFA (monounsaturated fatty acids), and SFA (saturated fatty acids).

The bioavailability of omega-3 fatty acids is influenced by their positional distribution within the acylglycerol, specifically between the sn-2 and sn-1,3 positions. Omega-3 fatty acids in the sn-2 position exhibit higher bioavailability compared to those in the sn-1,3 positions [48]. Supplementation with EPA in the sn-2 position has been shown to enhance the brain levels of EPA and DHA, offering a potential therapeutic approach for brain and mental health conditions [49]. In this study, we utilized ^13^C NMR spectroscopy to investigate the positional distribution of omega-3 fatty acids in the squid viscera oil obtained after lipase treatment. The carbonyl region of the resulting ^13^C NMR spectra was specifically analyzed (Figure 3), as it encompasses the distribution of key omega-3 fatty acids such as EPA and DHA [50,51].

As presented in Table 2, ETA is predominantly (>78%) positioned at the sn-1,3 locations, whereas MUFA, DPA, and SDA exhibit a more randomized distribution, with 44–61% located at the sn-1,3 positions and 38–56% at the sn-2 position. SFA is distributed at a 1:1 ratio over sn-2 and sn-1,3. EPA is preferentially distributed, with 72.5% at the sn-2 position and 27.5% at the sn-1 position. A similar preferential enrichment of EPA at the sn-2 position has been observed in oils derived from tuna [52], shrimp [53], and squid [54], as well as in lipase-treated salmon oil [55]. In contrast, DHA is predominantly found at the sn-2 position, with 92.9% of the total DHA at sn-2 and only 7.1% at the sn-1,3 positions. This predominant placement enhances its bioavailability and oxidative stability and is consistent with the position of DHA in naturally acylglyceride marine oils. For example, DHA is predominantly located at the sn-2 position in the triacylglycerides (TAGs) of salmon, mackerel, cod liver, and herring, while EPA tends to be more randomly distributed in these oils, as well as in those derived from hoki [50].

### 2.4. Rancimat Analysis, Lipid Oxidation Kinetics, and Shelf Life Prediction

**Figure 4:**
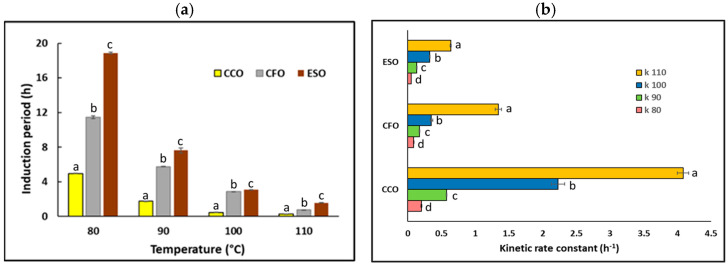
(**a**) Induction period (IP) of the oils against temperature (CCO—commercial calamari oil; CFO—commercial fish oil; ESO—enzymatically re-esterified squid output oil); (**b**) kinetic rate constant (k) of lipid oxidation of the CCO, CFO, and SE. Results are presented as mean ± SD. The bars not sharing common lowercase letters (labelled on the top of each bar) for each temperature in (**a**) and each oil in (**b**) are significantly different (*p* < 0.05).

The oxidative stability of the enzymatically converted squid visceral oil (ESO) studied at four different temperatures was compared to that of commercial calamari oil (CCO) and commercial fish oil (CFO), as shown in Figure 4a.

Crude squid oil showed induction periods of less than 0.1 h at all studied temperatures, which can be attributed to the high free fatty acid content (44%), making this oil very susceptible to autooxidation (with an induction period of 0.06 ± 0.01 h for FFA 44.5 ± 0.4% at 80 °C and 20 L/h air flow in Rancimat). Enzymatic re-esterification to convert most of the free fatty acid to acylglyceride produced a more stable oil (ESO) at tested temperatures (slightly higher at 100 °C), which was also more stable than CCO and CFO. Oil stability decreased with temperature for all oils. After 54 h, FFA was 4.0 ± 0.5% and the induction period was 18.9 ± 0.1% h at 80 °C and 20 L/h air flow in the Rancimat. The induction period changed from 0.06 to 18.9 h when FFA decreased from 44.5 to 4%, which illustrates the importance of low FFA for squid oil stability.

Changes in the induction period (IP) were demonstrated using first-order kinetics. The kinetic rate constant (k) increased as a function of temperature (80–110 °C) (*p* < 0.05), which indicated an increased rate of oxidation with temperature (Figure 4b). The lipid oxidation rate was in the order CCO > CFO > ESO. The lowest values for ‘k’ were observed for ESO at all tested temperatures, indicating greater thermo-oxidative stability compared to CCO and CFO.

Another lipid oxidation kinetic parameter, the activation energy for lipid oxidation (E_a_), showed substantial variation amongst the oils (*p* < 0.05) (Table 3). E_a_ had a range between 94.15 to 116.95 kJ/mol, with a lower value for ESO than for CCO or CFO. E_a_ indicates the energy required for initial oxidation and correlates with the production of primary oxidation products [39]. A lower E_a_ value indicates a delayed onset of rancidity [36] and confirms ESO is more stable than CCO and CFO.

The thermodynamic parameters of lipid oxidation, including activation enthalpies (ΔH) and activation entropies (ΔS), are given in Table 3. The regression coefficients (R^2^ > 0.95) indicated good suitability to models describing how the temperature affects lipid oxidation. Overall, the positive values of change in activation enthalpies indicate the endothermic process requiring elevated energy to initiate the reaction. While activation enthalpies (ΔH) and entropies (ΔS) indicated that the ESO and CFO shared closer rates compared to CCO, ESO exhibited the slowest lipid oxidation. Nonetheless, the negative value of entropy couples with the positive activation enthalpy for ESO signals for a non-spontaneous reaction. Unlike CCO and CFO, ESO showed a negative value of ΔS, indicating the formation of an ordered activated complex desirable for improved oxidative stability [35,36,56].

The influence of heating on lipid oxidation can be expressed by a temperature acceleration factor (Q_10_) (Table 3) ranging between 2.31 and 2.82 in the current study, being the maximum for CCO and the minimum for ESO. The lower value of Q_10_ for ESO indicates a slower rate of oxidation with a 10 °C rise in temperature. Similarly, the temperature coefficient (T_coeff_) with the minimum value for ESO, −8.36 × 10^−2^ K^−1^, is also consistent with slower oxidation for ESO compared to other oils.

The application of Rancimat enables the rapid determination of oxidative stability at multiple temperatures, along with the application of Equation (3), which enables extrapolation to other temperatures [36,56]. The shelf life of oils (Table 3) in the order ESO > CFO > CCO (*p* < 0.05) was consistent with the kinetic parameters. The high shelf life of the ESO could be attributed to stabilization by the natural presence of astaxanthin antioxidant together with low levels of free fatty acids after enzymatic esterification [57].

### 2.5. Astaxanthin Content

The astaxanthin content in the dark-brown-colored ESO was found to be 194.1 ± 0.2 µg/g of lipid, whereas no astaxanthin was detected in either CCO or CFO. Most fish oils do not contain astaxanthin, and both CFO and CCO squid oil undergo bleaching during the refining process. Bleaching is known to remove colored components, including astaxanthin. Astaxanthin is a naturally occurring carotenoid with a potent antioxidant capacity 10 times higher than β-carotene and 300 times higher than α-tocopherol [30]. The astaxanthin structure consists of two terminal six-membered rings with hydroxyl and carbonyl groups, joined by a polyene chain with eleven conjugated carbon-carbon double bonds, which endow it with exceptional capacity for scavenging reactive oxygen species (ROS) [58]. Therefore, astaxanthin contributes to the improvement of the oxidative stability of oils by neutralizing free radicals and reactive oxygen species. Its incorporation into oils delays lipid oxidation by scavenging free radicals, interrupting the chain reactions that lead to oxidative degradation [32,59,60]. Furthermore, natural astaxanthin is widely utilized as a nutraceutical approved by the United States Food and Drug Administration (USFDA) owing to its diverse bioactivities, including antioxidant, anti-cancer, neuroprotective, cardioprotective, osteoprotective, hepatoprotective, anti-inflammatory, and anti-diabetic properties [19]. Therefore, astaxanthin-rich oils are used as specialty oils in nutraceutical applications [61]. Considering this, oil processing methods should aim to preserve natural astaxanthin to enhance oil stability and leverage its associated health benefits. A notable commercial example is krill oil, which undergoes processing methods that intentionally avoid bleaching to preserve astaxanthin, resulting in a stable and high-nutritional-value oil [50]. This study highlights the role of astaxanthin in improving the oxidative stability of oil. Our analysis of oxidation kinetics and thermodynamics revealed that squid visceral oil containing astaxanthin exhibits a delayed onset of oxidation compared to commercial oils, CCO and CFO, which lack astaxanthin. Thus, the astaxanthin-rich lipase-treated squid visceral oil (ESO) obtained in this study demonstrates potential suitability for nutraceutical applications due to both improved stability and health benefits associated with astaxanthin.

## 3. Materials and Methods

### 3.1. Materials

Crude squid visceral oil was procured from Mantzaris Fisheries, North Geelong, Victoria, Australia. Immobilized *Rhizomucor miehei* lipase (Lipozym RMIM) was purchased from Oppenheimer Pty Ltd., Victoria, Australia. Commercial calamari oil and commercial fish oil were purchased from the market and used before their date of expiry. All other chemicals used were of analytical grade unless otherwise specified.

### 3.2. Lipase Processing of Crude Squid Visceral Oil

The crude squid visceral oil was processed using an immobilized enzyme lipase, as previously described in laboratory-scale studies [44]. In the present study, the pilot-scale processing of crude squid visceral oil was undertaken. A custom-built pilot scale reactor (200 L) was loaded with crude squid oil and glycerol at a ratio of 3:1, with a known amount of immobilized enzyme and molecular sieves at the bottom of the reactor. The reactor contents were pumped from top to bottom. The reactor contents were recirculated at 500 mL/min, held at 50 °C, and stirred at 500 rpm. Processed samples were taken intermittently up to 54 h to check the free fatty acid (FFA) content of the enzyme-processed squid visceral output oil (ESO).

### 3.3. Free Fatty Acid Content (%) and Other Lipid Classes by Capillary Chromatography with a Flame Ionisation Detector (Iatroscan)

The oil samples were analyzed using capillary chromatography connected to a flame ionization detector (Iatroscan MK-6, Iatron Laboratories Inc., Tokyo, Japan) with the three replicates of the samples, as previously reported [62], with slight modifications. The instrument was set at an air flow rate of 2 L/min, a hydrogen flow rate of 160 mL/min, and a scanning speed of 0.5 min/scan. The oil samples were then spotted onto the precleaned chromarods and developed in a tank containing a solvent mixture of heptane:diethyl ether:acetic acid (30:8.5:0.1, *v*/*v*/*v*) for 22 min and analyzed in an Iatroscan. The FFA percentage content and other lipid classes were determined by integration using SIC-480 II software Version 1.0.

### 3.4. Analysis of Fatty Acid Composition Using Gas Chromatography with a Flame Ionisation Detector (GC-FID)

The fatty acids in the oil samples were converted to methyl esters before analysis using gas chromatography. The method of analysis required 10 mg of oil samples dissolved in 1 mL of toluene, followed by the addition of 0.2 mL of internal standard (5 µg/µL methyl nonadecanoate (Sigma–Aldrich, Melbourne, Australia) in toluene) and 0.2 mL (1 µg/µL 2,6-di-tert-butyl-4-methylphenol (butylated hydroxytoluene; BHT, Sigma–Aldrich) in toluene) as the antioxidant in each of the oil samples. Then, 2 mL of acidic methanol (prepared by adding 1 mL of acetyl chloride (Sigma–Aldrich) dropwise to 10 mL of methanol on ice) was added to each of the samples, mixed well, and incubated overnight at 50 °C in a sealed tube. The mixture in each of the tubes was cooled, and 5 mL of sodium chloride solution (5% *m*/*v*) was added. The fatty acid methyl esters were extracted twice with 5 mL of heptane, and the heptane layer was washed with 5 mL of potassium bicarbonate solution (2% *m*/*v*). The heptane layer was then dried over sodium sulfate, followed by rotary evaporation to remove hexane, and the samples were then taken for analysis [59,62].

The samples were analyzed using a previously reported method [63] with minor modifications. An Agilent 6890 gas chromatograph (GC) with a flame ionization detector (FID) (Agilent, Mulgrave, Australia), equipped with a BPX70 SGE column (30 m length × 0.25 mm column ID × 0.25 μm film thickness; Supelco, Sigma–Aldrich, Melbourne, Australia), was used to analyze the samples. The oven was set to run at a rate of 4 °C/min from 140 °C (5 min hold) to 220 °C (5 min hold) for a total run period of 30 min. Then, 1 μL of the sample solution was injected with a split ratio of 50:1 (injector temperature, 250 °C). Helium was employed as the carrier gas with a constant flow of 1.5 mL/min. The detector gases were 30 mL/min hydrogen, 400 mL/min air, and 30 mL/min nitrogen. ChemStation B.04.03 software was used to integrate the peak areas, which were corrected using theoretical relative FID response factors.

### 3.5. Positional Distribution of Omega-3 Fatty Acids in Lipase-Treated Squid Visceral Oil by ^13^C-Nuclear Magnetic Resonance (NMR)

^13^C NMR analysis was performed to determine the positional distribution of omega-3 fatty acids in lipase-treated squid visceral oil by following the previous method [64]. An oil sample (250 mg ± 0.01 mg) was dissolved in deuterated chloroform (600 μL) and transferred into an NMR tube (5 mm) for analysis. The ^13^C NMR spectra were acquired using a Bruker 400 MHz instrument (Bruker, Avance III HD, Billerica, MA, USA), employing the following acquisition conditions: 12,000 scans, a spectral width of 238 ppm, and an acquisition time of 1.36 s. The DHA (C22:6 sn-2) peak was assigned at 172.0497 ppm, with other peaks in the carbonyl region referenced to the DHA sn-2 spectra [50]. The quantification of fatty acids in the carbonyl region was performed based on the area percentage of fatty acids obtained from the integrator response of the NMR spectra using TopSpin (version 4.3.0, Bruker).

### 3.6. Rancimat Test and Lipid Oxidation Kinetics

The oil samples were exposed at higher temperatures (80 °C, 90 °C, 110 °C, and 110 °C) in a saturated air flow of 20 L/h using Rancimat (743 model, Metrohm, Switzerland). The stability of the oil samples was expressed as an induction period (IP) in hours.

A kinetic rate constant (k) for lipid oxidation was assessed as an inverse of the induction period (k = 1/IP, h^−1^). The activation energies (E_a_, kJ/mol) and frequency factors (A, h^−1^) for lipid oxidation in the oil samples were determined using the Arrhenius Equation (1), as shown below:

ln(k) = ln(A) − (E_a_/RT), (1)

where k is the kinetic rate constant (h^−1^) and R is the molar gas constant (8.314 J/mol K). The activation enthalpies (ΔH) and entropies (ΔS) of lipid oxidation in the oil samples were calculated using the activated complex theory (Equation (2)), as shown below:

ln (k/T) = ln (k_B_/h) + (∆S/R) − (∆H/RT), (2)

where k_B_ is the Boltzmann constant (1.380 × 10^−23^ J/K) and h is the Planck’s constant (6.63 × 10^−34^ J s). ΔH and ΔS were determined using the slope and intercept of Equation (2) [11].

### 3.7. Prediction of Shelf Life

The shelf life of the oil samples was predicted by plotting the natural logarithm of the induction period (IP) against the absolute temperature (K) using Equation (3):

ln (IP) = a (T) + b,(3)

where ‘a’ represents slope and ‘b’ indicates the intercept of Equation (3). The slope was used for the determination of the temperature coefficients (T_coeff_., K^−1^). The temperature acceleration factor (Q_10_ number) was calculated using the ratio of the induction period (IP) at T and T + 10. The semi-logarithmic plots were extrapolated for calculating the shelf life at 25 °C.

### 3.8. Astaxanthin Quantification

Astaxanthin quantification (µg/g of lipid) in the oil samples was carried out using the method by Takeungwongtrakul and Benjakul [65], with minor modifications. A 0.3% (*w*/*v*) of the oil sample was mixed with petroleum ether and incubated at room temperature for 30 min. After appropriate dilution, the absorbance of the sample was measured at 468 nm using a Cary series UV-Vis spectrophotometer (Agilent Technologies, Mulgrave, Australia).

### 3.9. Statistical Analysis

The experiments were performed in duplicate (Rancimat, GC-FID, and NMR) or triplicate (all other tests) with data expressed as mean values ± standard deviation. Statistically significant differences between the datasets were evaluated (*p* < 0.05) using analysis of variance (ANOVA) carried out with the help of Minitab (Release 21.4.2. for Microsoft Windows) and Microsoft Excel software 2016. All graphs were drawn with Microsoft Excel software 2016.

## 4. Conclusions

An immobilized lipase-based process was successfully used at the pilot scale (200 L) to reduce the naturally occurring high levels of free fatty acid in squid visceral oil to produce acylglyceride-rich squid oil. The produced acylglyceride squid oil was rich in astaxanthin and the omega-3 fatty acids EPA and DHA, with EPA preferentially distributed at the sn-2 position and DHA predominantly retained at the naturally occurring sn-2 position, which generally improved bioavailability and stability. Since traditional colorimetric methods (PV and PAV methods) are not accurate for colored oils, including those containing astaxanthin, the Rancimat method was applied for investigating the oxidative stability of the squid visceral oil, which was compared with two commercial omega-3-rich oils. The kinetic and thermodynamic oxidation parameters were determined from the Rancimat data. The enzymatically processed high-acylglyceride product oil exhibited a much slower oxidation rate compared to the unprocessed input oil, commercial fish oil, and commercial calamari oil. This enhanced stability was attributed to both the retention of natural astaxanthin and lower FFA levels in the oil following enzymatic processing.

Some limitations of the current method include (a) challenges in obtaining high levels of triacylglyceride versus mono- and diacylglycerides and (b) difficulty in determining oil quality due to the presence of astaxanthin, which is colored and interferes with the standard PV and PAV colorimetric testing. For (a), it is necessary to remove water as it forms during the reaction to drive the reaction forward, since it is an equilibrium reaction. This is done using a high vacuum but becomes more challenging as the scale increases. Furthermore, the reaction requires the oil and water to remain in an emulsion during contact with the enzyme bed, which is more challenging as the scale increases. The presence of astaxanthin means that Rancimat is a better method for measuring stability than standard colorimetric methods. New and improved methods for detecting low levels of oxidation in colored oils are required for astaxanthin-containing oils.

Overall, this investigation shows that squid visceral oil can be converted to a relatively stable, low free fatty acid oil containing high natural levels of the antioxidant astaxanthin and omega-3 fatty acids, with retention of DHA positional distribution. The lower FFA and high astaxanthin and omega-3 levels, together with the high stability and natural DHA positional distribution retained at sn-2, make this oil an excellent nutritional oil with potential in nutritional supplements or functional foods.

## Figures and Tables

**Table 1:** Fatty acid composition of squid visceral oil during lipase processing. The results are presented as mean ± SD. The columns not showing common lowercase subscript for each row of fatty acid categories are significantly different (*p* < 0.05).

Fatty Acids (%) (Average of Three Replicates)	Lipase-Processed Crude Squid Oil			
	0 h	2 h	10 h	54 h
Saturated fatty acids total	25.6 ± 0.0 _a_	24.4 ± 0.1 _b_	23.4 ± 0.1 _c_	22.9 ± 0.4 _c_
Monounsaturated fatty acids total	26.9 ± 0.1 _a_	27.4 ± 0.1 _b_	27.5 ± 0.0 _b_	27.8 ± 0.1 _c_
Eicosapentaenoic acid (EPA)	20.2 ± 0.0 _a_	20.2 ± 0.0 _a_	20.8 ± 0.0 _b_	20.7 ± 0.1 _b_
Docosahexaenoic acid (DHA)	20.8 ± 0.0 _a_	20.9 ± 0.1 _a_	21.4 ± 0.0 _b_	21.3 ± 0.1 _b_
Omega-3 total	43.0 ± 0.1 _a_	43.1 ± 0.1 _a_	44.2 ± 0.0 _b_	44.1 ± 0.2 _b_
Omega-6 total	4.5 ± 0.0 _a_	4.60 ± 0.0 _b_	4.7 ± 0.0 _c_	4.7 ± 0.0 _c_
Polyunsaturated fatty acids total	47.3 ± 0.1 _a_	47.5 ± 0.2 _a_	48.7 ± 0.0 _b_	48.5 ± 0.0 _c_

**Table 2:** Positional distribution of EPA and DHA within the acylglycerides of lipase-treated squid visceral oils. ^a^ Values represent the mean of three independent spectral analyses performed using ^13^C NMR. Each spectrum was acquired with 12,000 scans to ensure accuracy.

Position	Fatty Acid	Molar Percentage (%) ^a^
sn-2	EPA	17.5 ± 1.0
	DHA	12.0 ± 0.1
	SDA	13.7 ± 1.2
	DPA	16.8 ± 0.0
	ETA	2.9 ± 0.5
	MUFA	16.3 ± 1.4
	SFA	20.8 ± 0.7
sn-1,3	EPA	6.6 ± 1.1
	DHA	0.9 ± 0.1
	SDA	10.8 ± 0.2
	DPA	23.8 ± 0.4
	ETA	10.5 ± 0.0
	MUFA	25.9 ± 2.1
	SFA	21.4 ± 1.0

**Table 3:** Kinetic and thermodynamic parameters of oils oxidation and shelf life (IP_25_) prediction using Rancimat. The columns not sharing a common lowercase subscript for each parameter are significantly different (*p* < 0.05).

Parameters	ESO ^†^_a_	CCO ^†^_b_	CFO ^†^_c_
Arrhenius constant (A, h − 1)	(4.57 ± 0.03) × 10^12^_a_	(4.11 ± 0.16) × 10^16^_b_	(4.52 ± 1.5) × 10^13^_c_
Activation energy for lipid oxidation (Ea, kJ/mol)	94.15 ± 0.04 _a_	116.95 ± 0.04 _b_	99.76 ± 0.95 _c_
Activation enthalpies (ΔH, kJ/mol)	91.09 ± 0.04 _a_	113.89 ± 0.04 _b_	96.7 ± 0.95 _c_
Activation entropies (ΔS, J/mol K)	−12.6 ± 0.05 _a_	63.08 ± 0.34 _b_	5.96 ± 2.88 _c_
Temperature coefficient (T_coeff_ × 10^−2^, K^−1^)	−8.36 ± 0.0 _a_	−10.38 ± 0.0 _b_	−8.91 ± 0.0 _c_
Temperature acceleration factor (Q_10_)	2.31 ± 0.0 _a_	2.82 ± 0.0 _b_	2.44 ± 0.02 _c_
Shelf life (IP_25_, days) ^‡^	74.42 ± 1.13 _a_	58.93 ± 1.7 _b_	72.97 ± 1.86 _c_

^†^ Coefficient of determination > 0.99 and ^‡^ Coefficient of determination > 0.95.

## Data Availability

Data will be provided upon request.
